# Postoperative aesthetic and healing features of postectomy using three different surgical techniques: a randomized, prospective, and interdisciplinary analysis

**DOI:** 10.1590/0100-6991e-20213045

**Published:** 2021-09-14

**Authors:** LORENZO NUNES ANGIOLETTI, LUKA MENDONÇA MELO FAJARDO, ELONIR GOMES, ELIANE MAZZUCO DOS SANTOS

**Affiliations:** 1 - UNISUL - Universidade do Sul de Santa Catarina, Acadêmicos do Curso de Medicina - Tubarão - SC - Brasil; 2 - UNISUL - Universidade do Sul de Santa Catarina, Professora Mestre em Educação - Tubarão - SC - Brasil; 3 - UNISUL - Universidade do Sul de Santa Catarina, Professora Mestre em Saúde Coletiva - Tubarão - SC - Brasil

It was with extreme interest that we read the article “Postoperative aesthetic and healing features of postectomy using three different surgical techniques: a randomized, prospective, and interdisciplinary analysis”, published in the Journal of the Brazilian College of Surgeons^1^. The healing and aesthetic results are divergent mainly due to the large number of medical specialties able to perform this procedure, but clinical studies have shown the Plastibell® (PB) technique as being the best in terms of healing and the subcuticular (SC) technique as rendering the best aesthetic aspect, based on the scores assigned by specialists from three different medical fields (Pediatrics, Dermatology, and Plastic Surgery).

It is clear from the reading that the study could be revisited through more descriptive methods as to the evaluation of aesthetic and healing criteria, but the contribution is undeniable as it demonstrates the position of different medical areas regarding aesthetic and healing criteria of a surgical procedure. As seen, Plastic Surgery proved to be the most rigorous, with many scores below 3, showing the vision of a doctor who deals daily with the concept of “beauty” and with the judgment of patients who are there precisely in search of “perfection”. Pediatrics, on the other hand, appears as a specialty that is not as rigorous as for the esthetic and healing postoperative evolution, with the highest percentage of scores above 4 and 5 (90%)^1^. 

Regarding complications, paraphimosis caused by displacement of the plastic ring is the most frequent (41.8%), followed by bleeding (32.9%) and preputial stenosis (22.8%)^2^. Hemorrhages and paraphimosis are more common in postectomies performed with the Plastibell® device, and preputial stenosis occurs more frequently in circumcisions by the conventional technique. However, according to Talini et al.^2^, there was no statistically significant difference between the complications arising from the different surgical techniques used in their service. Thus, there are no contraindications for the use of the Plastibell® device as for complications, it being the surgical technique with better healing results^1^, second in aesthetics only to the subcuticular suture method.

The study by Falcão et al.¹ paves the way to new studies that highlight the importance of the healing and aesthetic process throughout the patients’ lives. Moreover, one must contemplate the impact that may be caused by any aesthetic damage and, consequently, the assessment of possible harm to the patient’s self-esteem depending on the surgical technique used, in addition to considering the frequency of each complication. The descriptive study by Costa Jr.³ reflects upon the management of surgical planning and the indicators of operative time demanded in the operating room according to the specialties involved, to make time management ideal. This even allows changes that seek to improve applicability by effectiveness of the surgical method and the general management of the hospital. 

Nevertheless, there is lack of literature reviews that determine the criteria evaluated by more descriptive methods. It is important to qualify the aesthetics aspect and the impact on patients’ lives, and how it can affect their self esteem. However, it is crucial to recognize the importance of the averages achieved by the conventional method with subcuticular suture, and the importance of the appraised study itself in bringing new insights that lead to improvements in various areas of medical management¹. These come from understanding the aesthetic concept for the various specialties, the most suitable technique for each case, and new ways to apply time management to circumcision to optimize the operating room management³.
